# Bis{2-[3-(dimethyl­ammonio)­propyl­imino­methyl-κ*N*]-6-meth­oxy­phenolato-κ*O* ^1^}bis­(thio­cyanato-κ*N*)nickel(II)

**DOI:** 10.1107/S1600536810038298

**Published:** 2010-10-02

**Authors:** Ling-Wei Xue, Gan-Qing Zhao, Yong-Jun Han, Li-Hua Chen, Qin-Long Peng

**Affiliations:** a College of Chemistry and Chemical Engineering, Pingdingshan University, Pingdingshan Henan 467000, People’s Republic of China

## Abstract

The asymmetric unit of the title complex, [Ni(NCS)_2_(C_13_H_20_N_2_O_2_)_2_], consists of two half-mol­ecules, both of which are completed by crystallographic inversion symmetry (Ni^2+^ site symmetry = 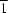 in both cases). Both metal ions are six-coordinated in distorted *trans*-NiO_2_N_4_ geometries arising from two *N*,*O*-bidentate Schiff base ligands and two *N*-bonded thio­cyanate ions. The mol­ecular conformations are reinforced by two intra­molecular N—H⋯O hydrogen bonds.

## Related literature

For related structures and background references, see: Xue *et al.* (2010*a* ▶,*b* ▶).
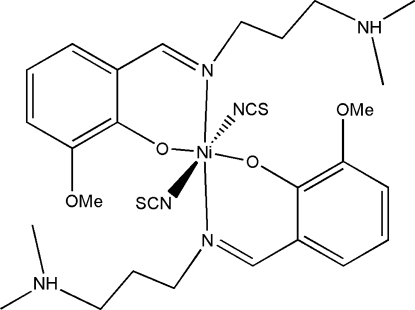


## Experimental

### 

#### Crystal data

[Ni(NCS)_2_(C_13_H_20_N_2_O_2_)_2_]
*M* *_r_* = 647.49
Monoclinic, 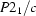

*a* = 16.228 (2) Å
*b* = 15.642 (2) Å
*c* = 13.3912 (18) Å
β = 113.132 (2)°
*V* = 3126.0 (7) Å^3^
*Z* = 4
Mo *K*α radiation
μ = 0.80 mm^−1^
*T* = 298 K
0.23 × 0.23 × 0.20 mm

#### Data collection

Bruker SMART CCD diffractometer
Absorption correction: multi-scan (*SADABS*; Sheldrick, 1996 ▶) *T*_min_ = 0.838, *T*_max_ = 0.857
17748 measured reflections
6772 independent reflections
4916 reflections with *I* > 2σ(*I*)
*R*_int_ = 0.026

#### Refinement

*R*[*F* ^2^ > 2σ(*F* ^2^)] = 0.035
*wR*(*F* ^2^) = 0.091
*S* = 1.02
6772 reflections
385 parameters
2 restraints
H atoms treated by a mixture of independent and constrained refinement
Δρ_max_ = 0.26 e Å^−3^
Δρ_min_ = −0.38 e Å^−3^

### 

Data collection: *SMART* (Bruker, 1998 ▶); cell refinement: *SAINT* (Bruker, 1998 ▶); data reduction: *SAINT*; program(s) used to solve structure: *SHELXS97* (Sheldrick, 2008 ▶); program(s) used to refine structure: *SHELXL97* (Sheldrick, 2008 ▶); molecular graphics: *SHELXTL* (Sheldrick, 2008 ▶); software used to prepare material for publication: *SHELXTL*.

## Supplementary Material

Crystal structure: contains datablocks global, I. DOI: 10.1107/S1600536810038298/hb5649sup1.cif

Structure factors: contains datablocks I. DOI: 10.1107/S1600536810038298/hb5649Isup2.hkl

Additional supplementary materials:  crystallographic information; 3D view; checkCIF report

## Figures and Tables

**Table 1:** Selected bond lengths (Å)

Ni1—O1	2.0403 (13)
Ni1—N3	2.0809 (18)
Ni1—N1	2.1102 (16)
Ni2—O3	2.0336 (14)
Ni2—N4	2.0990 (15)
Ni2—N6	2.1577 (19)

**Table 2:** Hydrogen-bond geometry (Å, °)

*D*—H⋯*A*	*D*—H	H⋯*A*	*D*⋯*A*	*D*—H⋯*A*
N2—H2⋯O1^i^	0.91 (1)	1.78 (1)	2.668 (2)	167 (3)
N5—H5*A*⋯O3^ii^	0.90 (1)	1.96 (2)	2.764 (2)	149 (2)

Symmetry codes: (i) 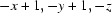; (ii) 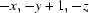.

**Fig. 1.**
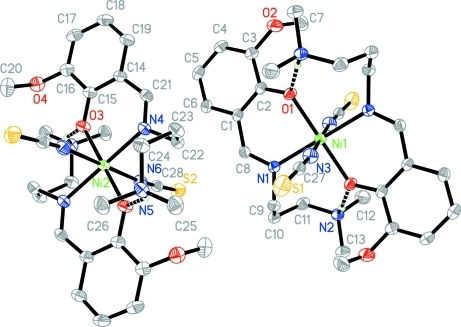
The structure of (I) with 30% probability displacement ellipsoids.

## supplementary crystallographic information

### Comment

Recently, we have reported a few Schiff base complexes (Xue *et al.*, 2010*a*,*b*). In this paper, a new nickel(II) complex with the Schiff base 2-[(3-dimethylammoniopropylimino)methyl]-6-methoxyphenol, is reported.

The complex is a centrosymmetric mononuclear nickel(II) complex, as shown in Fig. 1. The Ni atom, lying on the inversion center, is six-coordinated in an octahedral geometry, with two phenolate O and two imine N atoms from two Schiff base ligands defining the basal plane, and with two thiocyanate N atoms occupying the axial positions. The two amine N atoms are protonated, and form intramolecular N–H···O hydrogen bonds (Table 1) with the phenolate O atoms. The slight distortion of the octahedral coordination can be observed from the coordinate bond lengths and angles (Table 2).

### Experimental

3-Methoxysalicylaldehyde (152 mg, 1.0 mmol), *N,N*-dimethylpropane-1,3-diamine (102 mg, 1.0 mmol), ammonium thiocyanate (76 mg, 1.0 mmol), and nickel acetate tetrahydrate (249 mg, 1.0 mmol) were dissolved in methanol (80 ml). The mixture was stirred for two hours at room temperature. The resulting solution was left in air for a few days, yielding green blocks of (I).

### Refinement

H2 and H5A were located from a difference Fourier map and refined isotropically, with N–H distances restrained to 0.90 (1) Å. The remaining H atoms were placed in idealized positions and constrained to ride on their parent atoms with C–H distances of 0.93–0.97 Å, and with *U*_iso_(H) set at 1.2*U*_eq_(C) and 1.5*U*_eq_(C_methyl_).

### Crystal data

**Table:**

[Ni(NCS)_2_(C_13_H_20_N_2_O_2_)_2_]	*F*(000) = 1368
*M**_r_* = 647.49	*D*_x_ = 1.376 Mg m^−^^3^
Monoclinic, *P*2_1_/*c*	Mo *K*α radiation, λ = 0.71073 Å
Hall symbol: -P 2ybc	Cell parameters from 5211 reflections
*a* = 16.228 (2) Å	θ = 2.6–27.2°
*b* = 15.642 (2) Å	µ = 0.80 mm^−^^1^
*c* = 13.3912 (18) Å	*T* = 298 K
β = 113.132 (2)°	Block, green
*V* = 3126.0 (7) Å^3^	0.23 × 0.23 × 0.20 mm
*Z* = 4	

### Data collection

**Table:**

Bruker SMART CCD diffractometer	6772 independent reflections
Radiation source: fine-focus sealed tube	4916 reflections with *I* > 2σ(*I*)
graphite	*R*_int_ = 0.026
ω scans	θ_max_ = 27.0°, θ_min_ = 1.9°
Absorption correction: multi-scan (*SADABS*; Sheldrick, 1996)	*h* = −18→20
*T*_min_ = 0.838, *T*_max_ = 0.857	*k* = −19→19
17748 measured reflections	*l* = −17→7

### Refinement

**Table:**

Refinement on *F*^2^	Primary atom site location: structure-invariant direct methods
Least-squares matrix: full	Secondary atom site location: difference Fourier map
*R*[*F*^2^ > 2σ(*F*^2^)] = 0.035	Hydrogen site location: inferred from neighbouring sites
*wR*(*F*^2^) = 0.091	H atoms treated by a mixture of independent and constrained refinement
*S* = 1.02	*w* = 1/[σ^2^(*F*_o_^2^) + (0.0418*P*)^2^ + 0.616*P*] where *P* = (*F*_o_^2^ + 2*F*_c_^2^)/3
6772 reflections	(Δ/σ)_max_ = 0.001
385 parameters	Δρ_max_ = 0.26 e Å^−^^3^
2 restraints	Δρ_min_ = −0.38 e Å^−^^3^

### Special details

**Table:**

Geometry. All e.s.d.'s (except the e.s.d. in the dihedral angle between two l.s. planes) are estimated using the full covariance matrix. The cell e.s.d.'s are taken into account individually in the estimation of e.s.d.'s in distances, angles and torsion angles; correlations between e.s.d.'s in cell parameters are only used when they are defined by crystal symmetry. An approximate (isotropic) treatment of cell e.s.d.'s is used for estimating e.s.d.'s involving l.s. planes.
Refinement. Refinement of *F*^2^ against ALL reflections. The weighted *R*-factor *wR* and goodness of fit *S* are based on *F*^2^, conventional *R*-factors *R* are based on *F*, with *F* set to zero for negative *F*^2^. The threshold expression of *F*^2^ > σ(*F*^2^) is used only for calculating *R*-factors(gt) *etc*. and is not relevant to the choice of reflections for refinement. *R*-factors based on *F*^2^ are statistically about twice as large as those based on *F*, and *R*- factors based on ALL data will be even larger.

### Fractional atomic coordinates and isotropic or equivalent isotropic displacement parameters (Å^2^)

**Table:**

	*x*	*y*	*z*	*U*_iso_*/*U*_eq_	
Ni1	0.5000	0.5000	0.0000	0.03134 (10)	
Ni2	0.0000	0.5000	0.0000	0.03281 (10)	
S1	0.28158 (5)	0.31526 (5)	−0.26156 (5)	0.0710 (2)	
S2	0.18778 (4)	0.75758 (4)	0.05566 (5)	0.05524 (17)	
O1	0.48274 (9)	0.43998 (8)	0.12568 (11)	0.0399 (3)	
O2	0.54271 (11)	0.39569 (10)	0.33999 (13)	0.0592 (4)	
O3	0.01284 (9)	0.51671 (8)	0.15603 (11)	0.0395 (3)	
O4	−0.02789 (11)	0.52191 (10)	0.33375 (13)	0.0528 (4)	
N1	0.38379 (10)	0.57261 (10)	−0.02589 (13)	0.0341 (4)	
N2	0.50658 (12)	0.73003 (11)	−0.11985 (15)	0.0408 (4)	
N3	0.41756 (12)	0.41330 (11)	−0.11307 (15)	0.0452 (4)	
N4	0.11950 (10)	0.42923 (10)	0.05634 (13)	0.0347 (4)	
N5	0.00617 (11)	0.31873 (10)	−0.21919 (14)	0.0398 (4)	
N6	0.08080 (12)	0.61079 (11)	0.00524 (15)	0.0443 (4)	
C1	0.37093 (12)	0.53225 (12)	0.14375 (15)	0.0328 (4)	
C2	0.44253 (13)	0.47353 (12)	0.18471 (16)	0.0337 (4)	
C3	0.46936 (14)	0.44942 (13)	0.29512 (17)	0.0408 (5)	
C4	0.42342 (15)	0.47622 (14)	0.35629 (17)	0.0441 (5)	
H4	0.4417	0.4577	0.4278	0.053*	
C5	0.35069 (15)	0.53011 (15)	0.31371 (18)	0.0456 (5)	
H5	0.3195	0.5473	0.3556	0.055*	
C6	0.32498 (13)	0.55794 (13)	0.20875 (17)	0.0395 (5)	
H6	0.2762	0.5946	0.1798	0.047*	
C7	0.62574 (17)	0.4410 (2)	0.3822 (2)	0.0724 (8)	
H7A	0.6307	0.4765	0.3264	0.109*	
H7B	0.6744	0.4009	0.4067	0.109*	
H7C	0.6279	0.4760	0.4421	0.109*	
C8	0.34179 (13)	0.57118 (12)	0.03718 (16)	0.0357 (4)	
H8	0.2863	0.5982	0.0119	0.043*	
C9	0.34050 (14)	0.62316 (14)	−0.12656 (17)	0.0428 (5)	
H9A	0.2767	0.6115	−0.1568	0.051*	
H9B	0.3643	0.6052	−0.1793	0.051*	
C10	0.35530 (15)	0.71901 (14)	−0.10719 (19)	0.0491 (6)	
H10A	0.3274	0.7480	−0.1764	0.059*	
H10B	0.3246	0.7375	−0.0617	0.059*	
C11	0.45174 (15)	0.74753 (13)	−0.05485 (18)	0.0472 (5)	
H11A	0.4529	0.8085	−0.0412	0.057*	
H11B	0.4796	0.7192	0.0149	0.057*	
C12	0.60227 (16)	0.75222 (18)	−0.0561 (2)	0.0653 (7)	
H12A	0.6366	0.7408	−0.0990	0.098*	
H12B	0.6250	0.7184	0.0089	0.098*	
H12C	0.6069	0.8117	−0.0373	0.098*	
C13	0.47408 (16)	0.77696 (15)	−0.22516 (18)	0.0542 (6)	
H13A	0.4762	0.8374	−0.2117	0.081*	
H13B	0.4135	0.7602	−0.2683	0.081*	
H13C	0.5116	0.7635	−0.2634	0.081*	
C14	0.14218 (13)	0.42832 (12)	0.24865 (16)	0.0359 (5)	
C15	0.06605 (13)	0.47581 (12)	0.24231 (16)	0.0361 (5)	
C16	0.04936 (15)	0.48005 (13)	0.33849 (17)	0.0424 (5)	
C17	0.10847 (17)	0.44819 (14)	0.43617 (18)	0.0512 (6)	
H17	0.0965	0.4543	0.4983	0.061*	
C18	0.18630 (16)	0.40673 (14)	0.44204 (18)	0.0517 (6)	
H18	0.2275	0.3867	0.5082	0.062*	
C19	0.20132 (14)	0.39593 (13)	0.34964 (17)	0.0453 (5)	
H19	0.2521	0.3663	0.3533	0.054*	
C20	−0.10592 (18)	0.46990 (19)	0.2910 (2)	0.0693 (8)	
H20A	−0.1103	0.4453	0.2234	0.104*	
H20B	−0.1580	0.5042	0.2790	0.104*	
H20C	−0.1022	0.4251	0.3416	0.104*	
C21	0.16576 (13)	0.41259 (12)	0.15670 (17)	0.0376 (5)	
H21	0.2213	0.3873	0.1723	0.045*	
C22	0.16275 (13)	0.40526 (13)	−0.01820 (17)	0.0407 (5)	
H22A	0.2264	0.4171	0.0166	0.049*	
H22B	0.1383	0.4403	−0.0831	0.049*	
C23	0.14924 (14)	0.31075 (14)	−0.05085 (18)	0.0461 (5)	
H23A	0.1805	0.2991	−0.0980	0.055*	
H23B	0.1772	0.2764	0.0141	0.055*	
C24	0.05311 (14)	0.28187 (13)	−0.10793 (17)	0.0448 (5)	
H24A	0.0520	0.2200	−0.1135	0.054*	
H24B	0.0203	0.2977	−0.0639	0.054*	
C25	0.04371 (16)	0.28787 (17)	−0.2978 (2)	0.0575 (6)	
H25A	0.0398	0.2267	−0.3023	0.086*	
H25B	0.1053	0.3049	−0.2736	0.086*	
H25C	0.0102	0.3121	−0.3680	0.086*	
C26	−0.09173 (14)	0.30137 (17)	−0.26210 (19)	0.0568 (6)	
H26A	−0.1017	0.2408	−0.2647	0.085*	
H26B	−0.1199	0.3248	−0.3339	0.085*	
H26C	−0.1168	0.3274	−0.2154	0.085*	
C27	0.36096 (14)	0.37389 (13)	−0.17575 (16)	0.0374 (5)	
C28	0.12526 (14)	0.67114 (13)	0.02399 (16)	0.0378 (5)	
H2	0.5041 (18)	0.6730 (7)	−0.132 (2)	0.080*	
H5A	0.0166 (17)	0.3749 (7)	−0.207 (2)	0.080*	

### Atomic displacement parameters (Å^2^)

**Table:**

	*U*^11^	*U*^22^	*U*^33^	*U*^12^	*U*^13^	*U*^23^
Ni1	0.0356 (2)	0.02943 (18)	0.03020 (19)	−0.00061 (14)	0.01426 (15)	−0.00052 (14)
Ni2	0.03240 (19)	0.03016 (18)	0.02986 (19)	0.00455 (14)	0.00575 (15)	−0.00108 (14)
S1	0.0568 (4)	0.1032 (6)	0.0513 (4)	−0.0330 (4)	0.0194 (3)	−0.0272 (4)
S2	0.0585 (4)	0.0471 (3)	0.0575 (4)	−0.0116 (3)	0.0200 (3)	0.0014 (3)
O1	0.0518 (9)	0.0349 (7)	0.0406 (8)	0.0074 (6)	0.0264 (7)	0.0048 (6)
O2	0.0665 (11)	0.0570 (10)	0.0538 (10)	0.0182 (9)	0.0232 (9)	0.0186 (8)
O3	0.0434 (8)	0.0371 (7)	0.0321 (7)	0.0099 (6)	0.0084 (6)	−0.0007 (6)
O4	0.0635 (11)	0.0478 (9)	0.0506 (10)	0.0036 (8)	0.0261 (8)	−0.0062 (7)
N1	0.0351 (9)	0.0340 (8)	0.0331 (9)	−0.0007 (7)	0.0133 (7)	0.0008 (7)
N2	0.0445 (10)	0.0334 (9)	0.0467 (10)	0.0033 (8)	0.0204 (9)	0.0088 (8)
N3	0.0477 (11)	0.0399 (10)	0.0444 (10)	−0.0053 (8)	0.0141 (9)	−0.0058 (8)
N4	0.0327 (9)	0.0303 (8)	0.0365 (9)	0.0017 (7)	0.0087 (7)	−0.0034 (7)
N5	0.0380 (9)	0.0345 (9)	0.0427 (10)	0.0024 (8)	0.0113 (8)	−0.0082 (8)
N6	0.0416 (10)	0.0398 (10)	0.0475 (11)	0.0034 (8)	0.0133 (9)	−0.0019 (8)
C1	0.0340 (10)	0.0334 (9)	0.0331 (10)	−0.0070 (8)	0.0153 (9)	−0.0046 (8)
C2	0.0366 (11)	0.0312 (9)	0.0362 (11)	−0.0070 (8)	0.0174 (9)	−0.0030 (8)
C3	0.0460 (12)	0.0382 (11)	0.0384 (12)	−0.0023 (9)	0.0168 (10)	0.0028 (9)
C4	0.0545 (14)	0.0480 (12)	0.0317 (11)	−0.0076 (11)	0.0189 (10)	0.0008 (9)
C5	0.0523 (14)	0.0513 (12)	0.0427 (13)	−0.0087 (11)	0.0288 (11)	−0.0066 (11)
C6	0.0360 (11)	0.0429 (11)	0.0424 (12)	−0.0048 (9)	0.0184 (10)	−0.0035 (10)
C7	0.0526 (16)	0.108 (2)	0.0479 (15)	0.0185 (16)	0.0110 (13)	−0.0012 (15)
C8	0.0307 (10)	0.0389 (11)	0.0377 (11)	0.0003 (8)	0.0136 (9)	−0.0002 (9)
C9	0.0349 (11)	0.0547 (13)	0.0362 (11)	0.0008 (10)	0.0110 (9)	0.0086 (10)
C10	0.0525 (14)	0.0508 (13)	0.0524 (14)	0.0144 (11)	0.0296 (11)	0.0155 (11)
C11	0.0639 (15)	0.0369 (11)	0.0480 (13)	0.0042 (10)	0.0297 (12)	0.0034 (10)
C12	0.0496 (14)	0.0718 (17)	0.0665 (17)	−0.0102 (13)	0.0141 (13)	0.0197 (14)
C13	0.0613 (15)	0.0538 (14)	0.0496 (14)	0.0080 (12)	0.0240 (12)	0.0166 (11)
C14	0.0361 (11)	0.0297 (10)	0.0343 (11)	−0.0036 (8)	0.0056 (9)	0.0014 (8)
C15	0.0415 (11)	0.0263 (9)	0.0331 (11)	−0.0025 (8)	0.0068 (9)	−0.0021 (8)
C16	0.0512 (13)	0.0325 (10)	0.0416 (12)	−0.0030 (9)	0.0162 (11)	−0.0034 (9)
C17	0.0718 (16)	0.0454 (13)	0.0335 (12)	−0.0090 (12)	0.0176 (12)	−0.0002 (10)
C18	0.0560 (14)	0.0461 (13)	0.0386 (13)	−0.0057 (11)	0.0028 (11)	0.0095 (10)
C19	0.0406 (12)	0.0386 (11)	0.0443 (13)	−0.0019 (9)	0.0035 (10)	0.0066 (10)
C20	0.0624 (17)	0.0702 (17)	0.086 (2)	−0.0055 (14)	0.0402 (16)	−0.0064 (16)
C21	0.0303 (10)	0.0284 (9)	0.0460 (12)	0.0010 (8)	0.0064 (9)	0.0015 (9)
C22	0.0343 (11)	0.0443 (12)	0.0393 (11)	0.0029 (9)	0.0098 (9)	−0.0033 (9)
C23	0.0415 (12)	0.0449 (12)	0.0443 (12)	0.0087 (10)	0.0086 (10)	−0.0061 (10)
C24	0.0496 (13)	0.0352 (11)	0.0458 (12)	0.0050 (10)	0.0145 (10)	−0.0024 (9)
C25	0.0553 (15)	0.0642 (16)	0.0575 (15)	0.0057 (12)	0.0269 (12)	−0.0116 (13)
C26	0.0411 (12)	0.0712 (16)	0.0525 (14)	−0.0069 (12)	0.0123 (11)	−0.0120 (13)
C27	0.0397 (11)	0.0398 (11)	0.0367 (11)	0.0031 (9)	0.0194 (10)	0.0008 (9)
C28	0.0386 (11)	0.0380 (11)	0.0357 (11)	0.0089 (9)	0.0134 (9)	0.0035 (9)

### Geometric parameters (Å, °)

**Table:**

Ni1—O1	2.0403 (13)	C7—H7B	0.9600
Ni1—O1^i^	2.0403 (13)	C7—H7C	0.9600
Ni1—N3^i^	2.0809 (18)	C8—H8	0.9300
Ni1—N3	2.0809 (18)	C9—C10	1.524 (3)
Ni1—N1	2.1102 (16)	C9—H9A	0.9700
Ni1—N1^i^	2.1102 (16)	C9—H9B	0.9700
Ni2—O3^ii^	2.0336 (14)	C10—C11	1.509 (3)
Ni2—O3	2.0336 (14)	C10—H10A	0.9700
Ni2—N4	2.0990 (15)	C10—H10B	0.9700
Ni2—N4^ii^	2.0990 (15)	C11—H11A	0.9700
Ni2—N6	2.1577 (19)	C11—H11B	0.9700
Ni2—N6^ii^	2.1577 (19)	C12—H12A	0.9600
S1—C27	1.630 (2)	C12—H12B	0.9600
S2—C28	1.643 (2)	C12—H12C	0.9600
O1—C2	1.316 (2)	C13—H13A	0.9600
O2—C3	1.386 (3)	C13—H13B	0.9600
O2—C7	1.428 (3)	C13—H13C	0.9600
O3—C15	1.306 (2)	C14—C19	1.410 (3)
O4—C16	1.394 (3)	C14—C15	1.415 (3)
O4—C20	1.422 (3)	C14—C21	1.447 (3)
N1—C8	1.277 (2)	C15—C16	1.418 (3)
N1—C9	1.481 (2)	C16—C17	1.376 (3)
N2—C12	1.489 (3)	C17—C18	1.394 (3)
N2—C13	1.490 (3)	C17—H17	0.9300
N2—C11	1.494 (3)	C18—C19	1.362 (3)
N2—H2	0.906 (10)	C18—H18	0.9300
N3—C27	1.150 (2)	C19—H19	0.9300
N4—C21	1.283 (2)	C20—H20A	0.9600
N4—C22	1.477 (3)	C20—H20B	0.9600
N5—C26	1.487 (3)	C20—H20C	0.9600
N5—C25	1.488 (3)	C21—H21	0.9300
N5—C24	1.497 (3)	C22—C23	1.533 (3)
N5—H5A	0.897 (10)	C22—H22A	0.9700
N6—C28	1.154 (3)	C22—H22B	0.9700
C1—C6	1.409 (3)	C23—C24	1.512 (3)
C1—C2	1.412 (3)	C23—H23A	0.9700
C1—C8	1.450 (3)	C23—H23B	0.9700
C2—C3	1.418 (3)	C24—H24A	0.9700
C3—C4	1.372 (3)	C24—H24B	0.9700
C4—C5	1.378 (3)	C25—H25A	0.9600
C4—H4	0.9300	C25—H25B	0.9600
C5—C6	1.371 (3)	C25—H25C	0.9600
C5—H5	0.9300	C26—H26A	0.9600
C6—H6	0.9300	C26—H26B	0.9600
C7—H7A	0.9600	C26—H26C	0.9600
			
O1—Ni1—O1^i^	180.00 (7)	C10—C9—H9B	109.1
O1—Ni1—N3^i^	87.54 (6)	H9A—C9—H9B	107.8
O1^i^—Ni1—N3^i^	92.46 (6)	C11—C10—C9	115.79 (18)
O1—Ni1—N3	92.46 (6)	C11—C10—H10A	108.3
O1^i^—Ni1—N3	87.54 (6)	C9—C10—H10A	108.3
N3^i^—Ni1—N3	180.00 (7)	C11—C10—H10B	108.3
O1—Ni1—N1	88.84 (6)	C9—C10—H10B	108.3
O1^i^—Ni1—N1	91.16 (6)	H10A—C10—H10B	107.4
N3^i^—Ni1—N1	92.66 (6)	N2—C11—C10	114.93 (18)
N3—Ni1—N1	87.34 (6)	N2—C11—H11A	108.5
O1—Ni1—N1^i^	91.16 (6)	C10—C11—H11A	108.5
O1^i^—Ni1—N1^i^	88.84 (6)	N2—C11—H11B	108.5
N3^i^—Ni1—N1^i^	87.34 (6)	C10—C11—H11B	108.5
N3—Ni1—N1^i^	92.66 (6)	H11A—C11—H11B	107.5
N1—Ni1—N1^i^	180.00 (7)	N2—C12—H12A	109.5
O3^ii^—Ni2—O3	180.000 (13)	N2—C12—H12B	109.5
O3^ii^—Ni2—N4	90.50 (6)	H12A—C12—H12B	109.5
O3—Ni2—N4	89.50 (6)	N2—C12—H12C	109.5
O3^ii^—Ni2—N4^ii^	89.50 (6)	H12A—C12—H12C	109.5
O3—Ni2—N4^ii^	90.50 (6)	H12B—C12—H12C	109.5
N4—Ni2—N4^ii^	180.0	N2—C13—H13A	109.5
O3^ii^—Ni2—N6	87.27 (6)	N2—C13—H13B	109.5
O3—Ni2—N6	92.73 (6)	H13A—C13—H13B	109.5
N4—Ni2—N6	87.02 (6)	N2—C13—H13C	109.5
N4^ii^—Ni2—N6	92.98 (6)	H13A—C13—H13C	109.5
O3^ii^—Ni2—N6^ii^	92.73 (6)	H13B—C13—H13C	109.5
O3—Ni2—N6^ii^	87.27 (6)	C19—C14—C15	119.7 (2)
N4—Ni2—N6^ii^	92.98 (6)	C19—C14—C21	116.57 (19)
N4^ii^—Ni2—N6^ii^	87.02 (6)	C15—C14—C21	123.70 (17)
N6—Ni2—N6^ii^	180.0	O3—C15—C14	124.64 (19)
C2—O1—Ni1	124.97 (12)	O3—C15—C16	118.99 (19)
C3—O2—C7	112.77 (19)	C14—C15—C16	116.35 (18)
C15—O3—Ni2	127.67 (13)	C17—C16—O4	119.5 (2)
C16—O4—C20	113.10 (18)	C17—C16—C15	122.4 (2)
C8—N1—C9	115.21 (16)	O4—C16—C15	118.04 (19)
C8—N1—Ni1	123.83 (13)	C16—C17—C18	120.0 (2)
C9—N1—Ni1	120.71 (13)	C16—C17—H17	120.0
C12—N2—C13	109.21 (17)	C18—C17—H17	120.0
C12—N2—C11	110.42 (18)	C19—C18—C17	119.2 (2)
C13—N2—C11	112.99 (17)	C19—C18—H18	120.4
C12—N2—H2	107.2 (17)	C17—C18—H18	120.4
C13—N2—H2	109.6 (18)	C18—C19—C14	122.0 (2)
C11—N2—H2	107.2 (17)	C18—C19—H19	119.0
C27—N3—Ni1	168.91 (17)	C14—C19—H19	119.0
C21—N4—C22	114.76 (16)	O4—C20—H20A	109.5
C21—N4—Ni2	124.16 (14)	O4—C20—H20B	109.5
C22—N4—Ni2	120.55 (12)	H20A—C20—H20B	109.5
C26—N5—C25	109.85 (17)	O4—C20—H20C	109.5
C26—N5—C24	111.28 (18)	H20A—C20—H20C	109.5
C25—N5—C24	112.90 (17)	H20B—C20—H20C	109.5
C26—N5—H5A	110.2 (17)	N4—C21—C14	127.99 (18)
C25—N5—H5A	110.1 (18)	N4—C21—H21	116.0
C24—N5—H5A	102.3 (18)	C14—C21—H21	116.0
C28—N6—Ni2	169.71 (18)	N4—C22—C23	112.64 (17)
C6—C1—C2	120.01 (18)	N4—C22—H22A	109.1
C6—C1—C8	116.18 (18)	C23—C22—H22A	109.1
C2—C1—C8	123.79 (18)	N4—C22—H22B	109.1
O1—C2—C1	123.56 (18)	C23—C22—H22B	109.1
O1—C2—C3	119.90 (18)	H22A—C22—H22B	107.8
C1—C2—C3	116.52 (18)	C24—C23—C22	115.85 (17)
C4—C3—O2	120.12 (19)	C24—C23—H23A	108.3
C4—C3—C2	121.65 (19)	C22—C23—H23A	108.3
O2—C3—C2	118.21 (19)	C24—C23—H23B	108.3
C3—C4—C5	121.2 (2)	C22—C23—H23B	108.3
C3—C4—H4	119.4	H23A—C23—H23B	107.4
C5—C4—H4	119.4	N5—C24—C23	114.12 (18)
C6—C5—C4	119.0 (2)	N5—C24—H24A	108.7
C6—C5—H5	120.5	C23—C24—H24A	108.7
C4—C5—H5	120.5	N5—C24—H24B	108.7
C5—C6—C1	121.4 (2)	C23—C24—H24B	108.7
C5—C6—H6	119.3	H24A—C24—H24B	107.6
C1—C6—H6	119.3	N5—C25—H25A	109.5
O2—C7—H7A	109.5	N5—C25—H25B	109.5
O2—C7—H7B	109.5	H25A—C25—H25B	109.5
H7A—C7—H7B	109.5	N5—C25—H25C	109.5
O2—C7—H7C	109.5	H25A—C25—H25C	109.5
H7A—C7—H7C	109.5	H25B—C25—H25C	109.5
H7B—C7—H7C	109.5	N5—C26—H26A	109.5
N1—C8—C1	127.30 (18)	N5—C26—H26B	109.5
N1—C8—H8	116.3	H26A—C26—H26B	109.5
C1—C8—H8	116.3	N5—C26—H26C	109.5
N1—C9—C10	112.56 (17)	H26A—C26—H26C	109.5
N1—C9—H9A	109.1	H26B—C26—H26C	109.5
C10—C9—H9A	109.1	N3—C27—S1	178.0 (2)
N1—C9—H9B	109.1	N6—C28—S2	177.5 (2)

Symmetry codes: (i) −*x*+1, −*y*+1, −*z*; (ii) −*x*, −*y*+1, −*z*.

### Hydrogen-bond geometry (Å, °)

**Table:**

*D*—H···*A*	*D*—H	H···*A*	*D*···*A*	*D*—H···*A*
N2—H2···O1^i^	0.91 (1)	1.78 (1)	2.668 (2)	167 (3)
N5—H5A···O3^ii^	0.90 (1)	1.96 (2)	2.764 (2)	149 (2)

Symmetry codes: (i) −*x*+1, −*y*+1, −*z*; (ii) −*x*, −*y*+1, −*z*.
